# Gut Microbiome and Degradation Product Formation during Biodegradation of Expanded Polystyrene by Mealworm Larvae under Different Feeding Strategies

**DOI:** 10.3390/molecules26247568

**Published:** 2021-12-14

**Authors:** Emmanouil Tsochatzis, Ida Elizabeth Berggreen, Francesca Tedeschi, Konstantina Ntrallou, Helen Gika, Milena Corredig

**Affiliations:** 1 Department of Food Science, Aarhus University, Agro Food Park 48, 8200 Aarhus, Denmark; mc@food.au.dk; 2 CiFOOD—Centre for Innovative Food Research, Aarhus University, Agro Food Park 48, 8200 Aarhus, Denmark; 3 Department of Animal Science, Aarhus University, Blichers Alle 20, Tjele, Foulum, 8830 Viborg, Denmark; ideb@anis.au.dk; 4 Department of Molecular Biology and Genetics, Aarhus University, Gustav Wieds Vej 10, 8000 Aarhus, Denmark; f.tedeschi81@gmail.com; 5 FoodOmicsGR Research Infrastructure, AUTh Node, Center for Interdisciplinary Research and Innovation (CIRI-AUTH), Balkan Center B1.4, 10th Km Thessaloniki-Thermi Rd, P.O. Box 8318, 57001 Thessaloniki, Greece; kondrallou@gmail.com (K.N.); gkikae@auth.gr (H.G.); 6 School of Medicine, Aristotle University of Thessaloniki, 54124 Thessaloniki, Greece

**Keywords:** biodegradation of polystyrene (PS), insects, *Tenebrio molitor* larvae, gut microbiome, degradation compounds

## Abstract

Polystyrene (PS) is a plastic polymer extensively used for food packaging. PS is difficult to decompose and has low recycling rates, resulting in its accumulation in the environment, in the form of microplastic particles causing pollution and harming oceans and wildlife. Degradation of PS by mealworms (*Tenebrio molitor*) has been suggested as a possible biological strategy for plastic contamination; however, the biodegradation mechanism of PS by mealworms is poorly understood. It is hypothesized that the gut microbiome plays an important role in the degradation of PS by mealworms. This study carried out a comparative analysis of the gut microbiome of *Tenebrio molitor* larvae under different feeding strategies, and of the formation of degradation compounds (monomers, oligomers). A diet of bran:PS at 4:1 and 20:1 ratios was tested. The diet with the low ratio of bran:PS led to the presence of higher amounts of these compounds, compared to that with the high ratio. In addition, it was demonstrated that the addition of H_2_O significantly improved the biodegradation of PS monomer and oligomer residues, which could be identified only in the frass. The protein and nitrogen contents in insects’ biomass and frass varied amongst treatments. The diets resulted in differences in the gut microbiota, and three potential bacterial strains were identified as candidates involved in the biodegradation of PS.

## 1. Introduction

Plastic packaging is often associated with potential harm to the environment and human health [1]. Polystyrene (PS) is a plastic polymer extensively used for the production of food packaging contact materials (FCMs) due to its low cost, durability and mechanical properties [2]. Its large production and low recycling rates result in high potential for its accumulation in the environment, with risks for the survival of wildlife and detrimental effects on human health [3,4].

There is an increasing interest in the biodegradation of plastics, by microbial activity or specific enzymatic activity. In particular, studies have reported the potential degradation of PS using different insects: mealworms (*Tenebrio molitor*) [4,5,6,7,8,9,10], superworms (*Zophobas morio*) [11] and waxworms (*Galleria mellonella*) [3]. Biodegradation by insects has also been reported for other polymers such as polyethylene (PE) [12,13]. Metabolomics studies have revealed the presence of several bioactive compounds, in both the bulk insects’ biomass and frass, such as long-chain fatty acids, esters and amides. Furthermore, it has been shown that alteration in feeding affects the ratio of these compounds, with several long-chain acids, in addition to hydrocarbons, present in connection with the presence of PS and its degradation [4,6,7]. The addition of PS to the diet causes the insects to experience metabolic stress, in comparison to a control diet [4,6,7].

During PS biodegradation, increased formation and accumulation of long-chain free fatty acids (FFAs) in insects’ biomass [3] have been observed. The generation of FFAs during this process can be attributed to enzymatic degradation and increased metabolism within insects’ intestinal microbiota [4,14] and indicates digestion and biodegradation of PS [3]. Formation of amides has also been reported to correlate with the presence of enzymatic activity and bio-catalytic amide bond formation [7,15].

Brandon et al. demonstrated that mealworms’ gut microbiome contributes to accelerated plastic biodegradation [5], while others [16] highlighted that dominant phyla consist of *Proteobacteria*, *Bacteroides*, *Firmicutes* and *Actinobacteria*, and that predominant enzymes acting during the biodegradation of plastics are phosphatases, esterases, leucine arylamidase, β-galactosidase, β-glucuronidase, α-glucosidase, β-glucosidase, chitinase, α-mannosidase and α-fucosidase [16]. It has been indicated that the most abundant bacteria in *T. molitor* fed with PS were *Enterococcus* spp., *Listeria* spp., *Nitrospira defluvii* and bacteria belonging to *Propionibacterium*, *Streptococcus*, *Haemophilus*, *Staphylococcus*, *Lactobacillus*, *Pseudomonas* and *Clostridium* [6,17].

The scope of this research was to investigate changes in the gut microbiome of *Tenebrio molitor* larvae, fed PS-containing diets, with the aim to characterize its function during the biodegradation of PS in relation to the formation of PS monomers and oligomers as well as the growth, survival and PS consumption by the insects. Therefore, three different feeding protocols were studied and related to gut microbiota patterns during biodegradation.

## 2. Results and Discussion

### 2.1. Biodegradation of PS and Next-Generation Sequencing of Gut Microbiome under Different Feeding Protocols

The degradation of PS per gram of larvae increased throughout the experimental period from 0.4 to 1.7 mg PS/g larvae/day, after 15 days of the experiment, following a previously applied study [4]. This is in full agreement with previous studies [4]. The diet that involved the addition of H_2_O (Diet 3) showed an initial lag phase, with a low degradation rate at the beginning of the experiment, following a higher rate after one week, tentatively explained by the development and growth of the respective gut microbiome. This low rate of degradation, followed by a rate increase, was attributed to a period of adaptation of the gut microbiome to the change of feed and alteration to the gut microbiota environment [4]. In the other two diets, where water was not included as an additional nutrient, this adaptation phase was not observed. Further, it has been previously demonstrated that a low bran-to-PS ratio provides higher PS consumption. In other words, when more bran is available, less PS is consumed, as bran is preferred to PS [4]. The highest extent of PS consumption, of about 23% (weight/initial weight) after 15 days, was achieved with Diet 1, with a ratio of bran:PS of 4:1 (m/m), but also by Diet 2, containing a bran-to-PS ratio of 20:1 (m/m) + H_2_O [4]. Although comparable degradation rates between Diets 1 and 2 were observed, the survival of insects was different, where the high bran:PS ratio without H_2_O (Diet 3) resulted in low survival rates, while the high bran:PS ratio in the presence of H_2_O (Diet 2) presented better growth together with negligible differences in the survival rates compared to Diet 1.

Table 1 illustrates the main microbial populations present depending on the diet. The total bacterial counts were lower in the case of the bran:PS ratio of 4:1 m/m diet (Diet 1) compared to the 20:1 ratio, without (Diet 3) or with H_2_O (Diet 2) (Table 1). The diets including PS showed a significant decrease in the bacterial counts compared to the control diet, suggesting a potential bacteriostatic effect caused by chemical degradation products, such as styrene or styrene oligomers.

Next-generation sequencing was used to explore the larval gut microbiome and to investigate the effect of PS diets on the microbial communities. The microflora isolated from the digestive tracts of mealworm larvae produced 356,050 reads. Surprisingly, the most abundant genus found in the *T. molitor* microbiomes in all the feeding variants, including the control, was *Spiroplasma* sp., which is generally considered as a pathogen bacterium in insects [18]. The comparative study showed that bacteria from the families of *Enterobacteriaceae* (*Erwinia olea*) and *Streptococcaceae* (*Lactococcus lactis* and *Lactococcus garviae*) were more abundant in the gut samples of *T. molitor* fed with PS compared with the control, rendering them three of the most probable candidates playing a role in the biodegradation of PS. Interestingly, one of the most abundant, *Lactococcus garviae*, has already been identified as a possible candidate for polystyrene degradation [19].

In the literature, it is already reported that a large distribution of styrene-degrading abilities exists among different classes of pro- and eukaryotic microorganisms, where several different classes of bacteria such as Actinobacteria (e.g., *Mycobacterium, Rhodococcus*), *Bacillus*, *clostridium*, α-proteobacteria (*Sphingomonas*, *Xanthobacter*, *Goordnia*, *Rhodococcous*) and γ-proteobacteria (*Enterobacter*, *Pseudomonas*, *Xanthomonas*) can contain different genera and strains [20,21]. The results of this study, regarding the biodegradation of PS, present similarities to the available literature [20,21,22,23].

Table 2 compares the family, genus and species of fungi identified for the three different diets. In the case of microbial activity, there seemed to be a change in the gut microbiota stimulated by the presence of the polymer. However, this was not the case for fungi (Table 2). The counts were low, and there was no clear relation with the presence of PS in the feed. The highest number of reads was found in the insects fed the 20:1 bran to PS diet (Diet 3, 114 counts) compared with the control (26 counts) or the 4:1 ratio (Diet 1; 33 counts). The lowest values were measured for the 20:1 diet with H_2_O (Diet 2; 8 counts) (Table 2). Based on Table 1, it can be tentatively inferred that the content of water in the diet, compared to the non-inclusion of H_2_O, affected the bacterial microbiota, while the opposite effect was noticed for molds or fungi, where the presence of water in the diet reduced their presence. The data indicate that three fungal species, *Sporobdomyces roseus*, *Alternaria alternata* and *Aspergillus versicolor*, are more abundant in the gut samples of *T. molitor* fed with PS compared with the control. The presence of *Alternaria* *alternata* confirms previous reports where the same species, together with a consortium of other fungi, was found to grow during polyethylene degradation [24]. Furthermore, *Aspergillus versicolor* has been previously associated with the biodegradation of poly(butylene succinate-co-butylene adipate) (PBSA) [25]. Although the current work shows only preliminary data, these results may suggest, for the first time, an activity of these two fungi in PS degradation.

### 2.2. Chemical and Statistical Analysis

The insects’ biomass was analyzed for the presence of PS monomers, oligomers or NIAS compounds [1]. The results show no PS monomers in the biomass, but a limited number of PS oligomers and compounds derived from the plastic packaging (NIAS). Identified compounds were acetophenone, styrene, 2,4,6-triphenyl-1-hexene, 1,3,5-triphenylcyclohexane, 2,4-diphenyl-1-butene, 1,1-diphenyl-ethylene, trans-1,2-diphenycyclobu-tane, 1a-phenyl-4e-(1-phenylethyl)-1,2,3,4-tetrahydronaphthalene (1a-tetralin) and 1e-phenyl-4e-(1-phenylethyl)-1,2,3,4-tetrahydronaphthalene (1e-tetralin).

The concentrations of these compounds were related to the presence of gut microbiome strains and the identified bacterial strains. The larvae fed with a lower amount of bran (Diet 1) presented higher amounts (μg/mg sample) of degraded compounds (Table 3) than the diets with a higher bran:PS ratio (Table 3). It may be pointed out that the presence of H_2_O facilitated the growth of specific microbial species such as *Lactococcus lactis*, *Gregarina*, *Weisella* and *Enterococcus faecium* (Table 1). However, this difference did not seem to change the target analytes’ composition. Nonetheless, it may be hypothesized that the higher presence of lactic acid bacteria may be due to their importance in regulating host immune systems, enhancing gut metabolic capacities and maintaining a balance in the gut microbiota [26].

**Figure 1:**
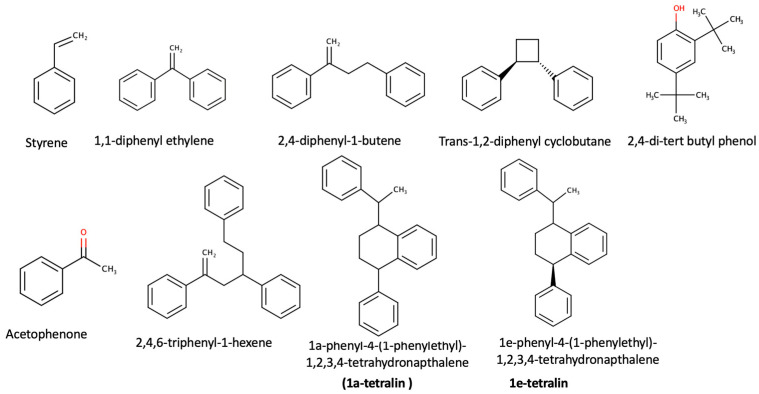
Chemical structures of identified monomers and side reaction products (acetophenone).

The chemical structures of the identified chemical compounds, listed in Table 3, are presented below in Figure 1.

Styrene can be oxidized to form acetophenone, which can be considered one of the basic side reaction products [7], while 2,4-di-tert butyl phenol is a degradation product of an additive, namely, Irgafos 168 [27]. All the remaining compounds are styrene oligomers that are degradation products of the PS polymer [1].

### 2.3. Multivariate Statistical Analysis

Several bacterial species were identified in mealworms’ gut microbiome. Multivariate modeling was carried out on microbiota and chemical data using principal component analysis (PCA) (Q2 = 0.821). The results are summarized in Figure 1 and combined with insect growth values, as well as measurements of nitrogen recovered in the frass and insect biomass, measured in a parallel study.

The control diet and Diet 1 had significantly higher values of nitrogen in the frass, compared to Diet 2 and Diet 3, where increased survival rates and growth were observed (Figure 1). Figure 1 demonstrates the clustering of the four diets. The control diet and Diet 3 (bran:PS 20:1, no water added) were clustered based on their gut microbiomes and their frass and insect (biomass) protein. The control diet presented the highest number of counts mainly for *Klebsiella* and *Spiroplasma* spp. The other two diets clearly clustered in the opposite quadrants. In the case of Diet 2 (20:1 + H_2_O), there was a clear differentiation in the growth of *Enterococcus faecium*, *Weisella* and *Lactobacillus lactis* along with *Gregarina polymorpha* (see also Table 1). Furthermore, in Diet 2 (including H_2_O), the growth of *T. molitor* was higher in this case and was related to higher survival rates, probably due to the better hydration and better health of the insects (Diet 2). In contrast, in Diet 3 (bran:PS ratio of 20:1 ratio without H_2_O), the average insect’s weight was higher, and the survival rates were reduced (more dead insects), most probably due to the pure hydration of the insects that reflected in higher consumption of bran and eventually a higher weight. However, higher weight does not necessarily correlate with high PS consumption. Moreover, it seemed that the growth of larvae is not necessarily translated to a better survival rate but to the idea that insects could have been less healthy and had a less healthy gut microbiome.

As the insects would prefer to consume bran rather than PS, an increased ingestion of bran results in a higher average weight gain. Figure 1 shows that the type of associated bacteria was different between the diets with a 20:1 ratio with (Diet 2) and without H_2_O (Diet 3). This can be correlated with different physiological differences (growth, weight) and may result in different degradation products. Diets 2 and 3 (20:1 bran:PS) also included higher amounts of bran, and they were related to a lower number of live insects/time and low PS consumption. Diet 3, however, clustered around another group of bacteria (*Chryseobacterium*, *Bacteroides*, *Clostridium*, *Spiroplasma*, *Chryseobacterium*), as well as having a higher average weight (reflecting the lower duplication rate). Again, this would be due to the higher availability of bran. Previously published works reported that the most abundant bacteria in *T. molitor* fed with PS were *Enterococcus* spp., *Listeria* spp., *Nitrospira defluvii* and bacteria belonging to *Propionibacterium*, *Streptococcus*, *Haemophilus*, *Staphylococcus*, *Lactobacillus*, *Pseudomonas* and *Clostridium* [6,17]. The present study therefore indicates that microbiome patterns may completely differ among studies within the same gut host, and that the role of the whole microbiota and the potential interactions existing between the bacterial communities living in the digestive tract of *T. molitor*, the environment and their role in PS biodegradation need to be further understood.

**Figure 2:**
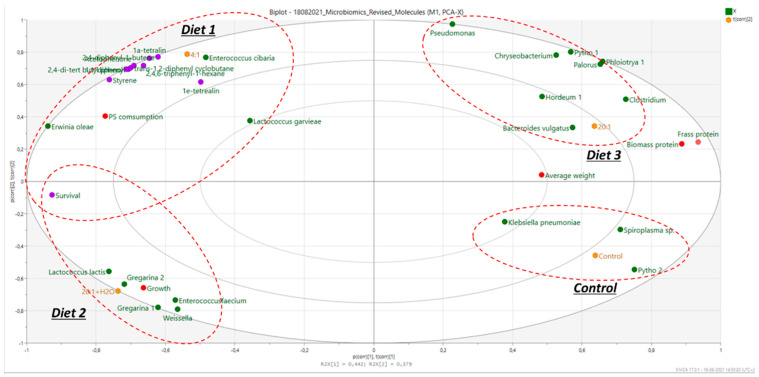
PCA biplot for the analysis of the applied diets in relation to the associated microbiome (purple dots: monomers/oligomers/plastic-oriented compounds; red dots: physical condition and protein/nitrogen of insects; green dots: microbial species).

The multivariate model also showed that two diets (control and bran:PS 20:1; Diet 3) clustered around a significant higher average weight, a higher presence of bacteria that do not support gut health (e.g., *Klebsiella*), increased protein content and frass nitrogen of the larvae and lower PS biodegradation yields. On the contrary, the amount of PS residues (monomers, PS oligomers) was higher in the case of the low bran:PS ratio (Diet 1; 4:1); this could be correlated with the different microbiomes and tentatively attributed to different enzymatic activities of the existing microbiome. Diet 2 also clustered closer to Diet 1, although differences were observed in relation to the amounts of monomers (styrene), degradation compounds and NIAS. Higher amounts were observed in the case of Diet 1 (bran:PS 4:1) compared to Diet 2 (20:1+ H_2_O) due to the potentially lower biodegradation of PS. It may be possible to hypothesize that the presence of H_2_O, together with a high bran:PS ratio, facilitated better PS consumption (left-hand side) (Figure 2) and presented higher survival rates while containing less NIAS and less degradation compounds (bottom left vs. top left-hand side). This could be attributed to better hydration, better health of insects and a better gut microbiome, as can be inferred by the presence of lactic acid bacteria (probiotics).

The diet that showed the most distinct bacteria cluster was that which contained a high ratio of bran:PS and water (20:1 + H_2_O; Diet 2). In this diet, the growth of the insects was higher, due to the presence of H_2_O, causing better hydration. Moreover, PS consumption was higher, likely due to the presence of a distinct microbiota, affecting larvae’s metabolism, as already reported [4].

In conclusion, the combination of a high-bran diet and proper hydration (presence of H_2_O) seems to support a better gut microbiome, higher PS consumption and higher survival rates, in contrast with only high bran amounts (Diet 3; bran:PS 20:1). Insects subjected to Diet 3 showed the development of anaerobic bacteria such as *Clostridium*, supporting earlier findings that this bacterium is stimulated by the presence of large amounts of nutrients that cannot be digested by the host. This also relates to the production of higher levels of short-chain fatty acids (SCFAs) [7], which play a noticeable role in intestinal homeostasis [28]. Indeed, with these diets (Diet 1; Diet 2), there was a reduced protein content in insects’ biomass and increased survival rates, most probably due to the presence of less bran (Diet 1) or to the presence of a higher bran content and H_2_O. This can be tentatively explained by the better physiological condition of the insects and the adaptation to nutritional stress [29,30,31].

From the PCA (Figure 1) and clustering analysis, it seems that the microbiomes of the control and 4:1 (Diet 1) diets were fairly identical and clustered well, due to the similarities of the bran content within the diet. Additionally, these results were directly correlated with high survival rates, which is crucial for the increase and evolution, either for the degradation of the plastic [4] or for the growth and reproduction of the larvae [8].

### 2.4. Metabolic Pathways for Styrene Biodegradation

**Figure 3:**
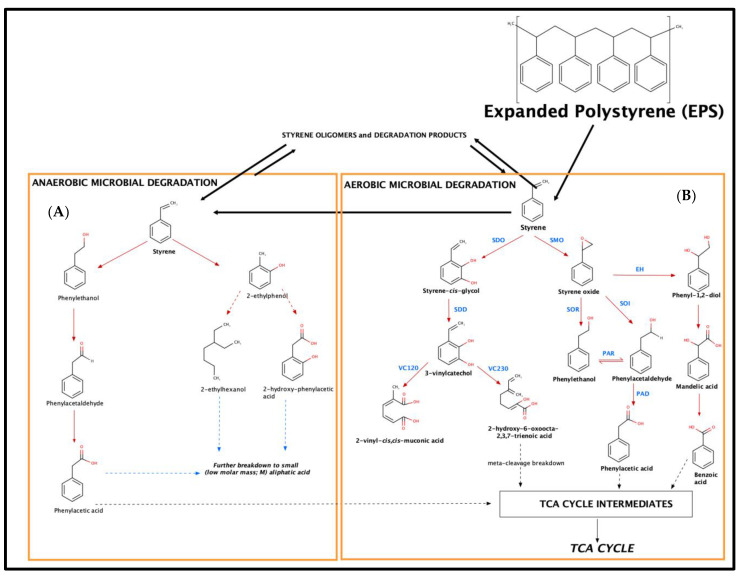
Identified degradation products and metabolic pathways for: (**A**) anaerobic degradation and (**B**) aerobic degradation (abbreviations: SMO—styrene monoxygenase; SOI—styrene oxide isomerase; SDO—styrene-2,3-dioxyxegenase; VC12O—vinylcatechol 1,2-dioxygenase; VC230—vinylcatechol 2,3-dioxygenase; SOI—styrene oxide isomerase; PAD—phenylacetaldehyde dehydrogenase; SOR—styrene oxide reductase; PAD—phenylacetaldehyde dehydrogenase) (adapted from [21,23,29]).

In the literature, two main metabolic pathways for the biodegradation of styrene by microorganisms have been proposed, referring to aerobic and anerobic degradation [20,21,23,29]. Both metabolic degradation pathways are presented schematically in Figure 3.

Within these two routes, oxygen, either molecular or coming from H_2_O, is used by the microorganism in order to activate styrene [21]. The first step involves the action on the aromatic nucleus or the vinyl sidechain [20]. Especially in the case of the vivid sidechain, it seems that aerobic microbiological epoxidation occurs [20]. Hence, for anaerobic degradation, the sidechain of styrene is oxidized by styrene monoxygenases (SMOs) to styrene oxide, while styrene-2,3-dioxyxegenase (SDO) introduces two oxygen atoms (hydroxy groups), forming styrene cis-glycol. Styrene-2,3-dihydrodiol dehydrogenase (SDD) catalyzes the re-aromatization to 3-vinylcatechol [20,21,22,23]. 3-Vinylcatechol might undergo meta-cleavage breakdown by vinylcatechol 1,2-dioxygenase (VC12O) or vinylcatechol 2,3-dioxygenase (VC23O), yielding 2-vinyl-cis,cis-muconic acid and 2-hydroxy-6-oxoocta-2,4,7-trienoic acid [21]. 2-Hydroxy-6-oxoocta-2,4,7-trienoic acid can lead to intermediate chemical compounds that can be introduced to the tricarboxylic acid (TCA) cycle and lead to degradation to CO_2_ and H_2_O [21].

From the other side, the formed reactive oxirane is then isomerized to phenylacetaldehyde by a styrene oxide isomerase (SOI). In contrast to cytosolic SMOs, several studies indicated a membrane-bound localization of SOIs in *Pseudomonas*, *Corynebacterium* (Itoh et al., 1997a) and *Rhodococcus* (unpublished; phenylacetaldehyde is then converted to phenylacetic acid by a phenylacetaldehyde dehydrogenase (PAD). It should be clarified that in this case, an equilibrium reaction exists among phenylethanol and phenylacetaldehyde (action of a phenylacetaldehyde reductase, PAR), which are formed from the action of a styrene oxide reductase (SOR), and a styrene oxide isomerase (SOI) [20,21,23]. Finally, the formed phenylacetaldehyde is then converted to phenylacetic acid by a phenylacetaldehyde dehydrogenase (PAD) that can be introduced to the tricarboxylic acid (TCA) cycle and lead to degradation to CO_2_ and H_2_O [21]. Finally, for aerobic degradation, another mechanism involves the biochemical hydrolysis of styrene oxide to phenylethan-1,2-diol, as reported by an epoxide hydrolase (EH), and oxidation to mandelic acid by a dehydrogenase [20,21,22,23] (Figure 3).

In terms of metabolic pathways in relation to anaerobic degradation of styrene, they initially involve the water-mediated hydroxylation of the aromatic ring, followed by a reduction of the vinyl sidechain to yield 2-ethylphenol, which may be exposed either to a ring cleavage (formation of 2-ethylhexanol) or a sidechain oxidation (formation of 2-hydroxyphenyl- acetic acid). The latter metabolic products, although not identified, lead to the formation of intermediate chemical compounds that can be introduced to the tricarboxylic acid (TCA) cycle and, finally, lead to degradation to CO_2_ and H_2_O [21]. Both aerobic and anaerobic degradation pathways seem to be important and to occur at the same time by different microbial consortia (Figure 3).

Finally, it must be highlighted that a reduced degradation might reflect in higher amounts of styrene monomers and oligomers on the biomass of the larvae, due to the insufficient growth of microbiological species. This is aligned with the low quantities of monomers and oligomers in the presence of H_2_O, where the microbiome could support the intense biodegradation of the polymer and eventually styrene, following existing mechanisms.

Additionally, the formation of oligomers and the action of the bacteria support the potential formation of styrene monomers. Therefore, besides styrene itself, microorganisms can metabolize or even use substituted styrene compounds or oligomers as sole sources of carbon and energy [21], indicating an equilibrium towards the formation of styrene (Figure 3).

From all these mechanisms and from the results presented in Section 2.3, it is clear that the presence of H_2_O is highly important not only to support the growth of appropriate bacteria and microbiota but also to act as an important source of oxygen. Oxygen is needed in order to support both aerobic and anaerobic metabolic degradation of styrene. Moreover, it has to be mentioned that the presence of H_2_O also supports the well-being and health of the larvae [4].

Moreover, the results are in accordance with a previous study [4], where a lower amount of monomers and oligomers was excreted to the environment through insects’ frass, where in the presence of H_2_O, much fewer mass fractions were found, confirming the growth of an efficient gut microbiome that led to appropriate biodegradation to CO_2_ and H_2_O, through an aerobic degradation mechanism (presence of oxygen from the H_2_O). This effect was also present in the presence of a higher amount of bran without water (Diet 3), where it was most probably supported by an anaerobic degradation mechanism. Regarding this study, the microbiome results presented previously can be correlated with the identified degradation compounds and with their quantities within the different feeding treatments. Our findings are in accordance with the existing styrene microbiological metabolic pathways as well as with the biotransformation of styrene-based degradation products, such as oligomers.

Finally, it has to be highlighted that the excretion of monomers and oligomers in the environment can be considered negligible and low, presuming a low source of pollution, as reported in a previous study, by excretion through insects’ frass [4]. However, the amount of these compounds should always be taken into consideration to consider the biochemical process efficient and complete, in terms of remediation

## 3. Materials and Methods

### 3.1. Test Materials and Tenebrio molitor Larvae

Expanded PS (EPS) foam packaging (density 0.014 g/cm^3^) was purchased at a local supermarket. PS material was assessed by attenuated total reflection-Fourier transform infrared (ATR-FTIR) spectroscopy and differential scanning calorimetry (DSC). Recorded spectra and thermograms were evaluated and confirmed the PS nature of the samples (matching FTIR spectra and a glass transition of 100 °C). *T. molitor* larvae were reared at Aarhus University, Department of Animal Science. Prior to the experiment, the larval diet consisted of rolled barley and a mixture of vegetables as a source of water. The composition of the bran is presented in Table 4.

### 3.2. Biodegradation of PS

In addition to the control diet of rolled barley, three experimental setups were compared, involving three diets. Diet 1 was based on previously published studies [6] and was chosen to ensure the comparability of the results. It was based on a bran:PS ratio of 4:1 *w*/*w* (Diet 1), where, in all cases, the amount of PS remained identical. However, because of the higher portion of unavailable nutrition from the shell of the grain, compared to wheat bran, an increased amount of feed was used to avoid starvation of *T. molitor* larvae. Water is known to have a large influence on larvae growth [32]. To test the effect of water on PS degradation by larvae, water was added to Diet 2 together with an increased bran:PS ratio, 20:1 *w*/*w* + H_2_O (1.5 mL every fourth day). This ratio was also applied with the absence of water as a diet (Diet 3), in order to investigate whether larvae were more or less prone to eat PS when more feed was present, together with or without the presence of water. Larvae survival and degradation of PS were assessed in 75 larvae atoms (weighting 78.0 ± 3.0 mg/larvae) that were placed into 50 cm^2^ cylindrical plastic polypropylene (PP) containers, with a density:surface of 1.5 larvae/cm^2^, in a climate-controlled room maintaining a temperature of 25 °C ± 1 °C and having a controlled relative humidity of 55 ± 5%. All the aforementioned biodegradation experiments were performed in duplicate, receiving identical amounts of PS plastic. In all experiments, 1000 mg ± 23 mg of PS plastic was provided to the larvae. In each experimental container, a specific amount of diets was provided, as described in Table 5. The PS was cut into 2–3 cm cubes. Insects’ survival rate was assessed by counting dead larvae every 4 days within a total of 15 days, while the dead larvae were reported and removed from the cylindrical container. At the end of the experiment, the larvae were collected, by sieving them, and stored at −80 °C until further analysis.

### 3.3. Collection of Microflora

To eliminate the microflora present on the external surface of the larvae, each larva was rolled on a thin layer of EtOH (2 mL of 70% EtOH in a standard Petri dish) for approximately 8 s. Using two tweezers, the bottom of the larva was broken by pulling out the intestine, and the larva was squeezed to allow all the remaining internal liquid to come out. The “skin” of the larva was discharged. For every intervention group, 20 larvae were used. The mixtures of larvae intestines and internal liquid were diluted in 3 mL of MRD liquid (Thermo Fisher; peptone 1.0 g/L, sodium chloride 8.5 g/L). The mixture was stirred, and the liquid was collected with a 2.5 mL syringe and placed into two cryotubes. The tubes were centrifuged for 30 s to allow removal of heavy debris.

### 3.4. DNA Extraction

For every sample, 150 µL of the supernatant was mixed with 260 µL NucliSENS^®^ Lysis Buffer (bioMérieux, Marcy-l’Etoile, France); the mixture was kept at room temperature for 48 h and was refrigerated at 5 °C until DNA extraction, which was performed within 4 days from sample collection. Samples were analyzed and sequenced within 4 days from sample collection, as previously described by Ring et al. [33]. Briefly, DNA extraction was carried out by mixing 260 μL of sample in tubes containing 1.4 mm zirconium beads (OPS Diagnostics LLC, Lebanon, NJ, USA) and bead beating in a TissueLyser II (QIAGEN, Hilden, Germany) for 2 min, followed by 8 min of incubation at room temperature, in order to release DNA. After centrifugation (16,000× *g* 5 min), 100 µL of the supernatant was processed by the automated EMAG^®^ Nucleic acid extraction system (bioMérieux, Marcy-l’Etoile, France) according to the manufacturer’s protocol.

### 3.5. 16S-18S Assay: NGS-Based Detection and Differentiation of Nuclear Ribosomal Genes

The three following primer sets were chosen for 18S rRNA genes: G3F1/G3R1 (GCCAGCAGCCGCGGTAATTC/ACATTCTTGGCAAATGCTTTCGCAG), G4F3/G4R3 (CAGCCGCGGTAATTCCAGCTC/GGTGGTGCCCTTCCGTCAAT) and G6F1/G6R1 (TGGAGGGCAAGTCTGGTGCC/ACGGTATCTGATCGTCTTCGATCCC). G3 and G6 primers target the hypervariable regions V3–V4, and G4 targets the hypervariable regions V3–V5 of the 18S rRNA gene. For 16S rDNA amplification, a modified version of the published universal prokaryotic primers 341F/806R, targeting the V3–V4 hypervariable regions, was used [34]. The forward primer had three additional nucleotides attached to the 5′ end (ACTCCTAYGGGRBGCASCAG, 341F3), and the reverse primer had five additional nucleotides attached to the 5′ end (AGCGTGGACTACNNGGGTATCTAAT, 806R5). For each primer pair, rDNA was amplified using a short PCR setup as follows: initial denaturation at 95 °C for 3 min was followed by 20 cycles of 95 °C (16S: for 30 s; 18S: for 1 min), 60 °C for 1 min and 72 °C for 30 s; final elongation was carried out at 72 °C (16S: for 7 min; 18S: for 4 min). PCR was performed in a 25 μL volume, using the Extract-N-Amp PCR ReadyMix (Sigma-Aldrich, St Louis, MO, USA) with 0.4 μM of each primer and 2 μL of template. This PCR is referred to as PCR1. The products from PCR1 were prepared for sequencing by a second PCR (PCR2) using the same PCR program. PCR2 attached an adaptor (A), an index (i5) and a forward sequencing primer site (FSP) to the 5′ end of the amplicons and an adaptor (B), an index (i7) and a reverse sequencing primer site (RSP) to the 3′ end of the amplicons. Hence, four PCR products were generated for each sample. DNA was quantified using the Quant-ITTM 121 dsDNA High Sensitive Assay Kit (Thermo Fisher Scientific, Carlsbad, CA, USA), and PCR2 products were pooled in equimolar amounts between samples into the pooled amplicon library (PAL). Undesirable DNA amplicons were removed from the PAL by using Agencourt AMPure XP bead (Beckman Coulter) in a two-step process. Firstly, DNA fragments below 300 nucleotides were removed by a 10 μL PAL-to-24 μL AMPure beads ratio, following the manufacturer’s protocol, and eluted in 40 μL TE buffer. Secondly, large DNA fragments above 1 kbp were removed by a 10 μL AM1-to-16 μL AMPure beads ratio. The resulting AMPure bead-purified PAL was denoted bPAL. bPAL was diluted to its final concentration of 11.5 pM DNA with a 0.001 N NaOH concentration and used for sequencing on the Illumina MiSeq (Illumina Inc., San Diego, CA, USA). The library was sequenced with the 500-cycle MiSeq Reagent Kit V2 in a 2 (x) 250nt setup (Illumina Inc., San Diego, CA, USA). Data analysis was performed using BION (http://box.com/bion, accessed on 7 June 2021). The pipeline accepts raw sequences and includes steps for de-multiplexing, primer extraction, sampling, sequence- and quality-based trimming and filtering, de-replication, clustering, chimera checking, reference data similarities and taxonomic mapping and formatting. Non-overlapping paired reads were allowed for analysis [19].

### 3.6. Nitrogen/Protein Content in Frass and Biomass

Crude protein content in insects’ biomass was determined by Dumatherm Nitrogen/Protein analyzer (Gerhardt, Nitrogen and Protein Analyser, Königswinter, Germany) using a protein-to-nitrogen conversion factor of 6.25. Calibration was performed with EDTA. The combustion reactor operated at 979 °C, the reduction reactor at 649 °C and the degassing oven at 299 °C. Data were analyzed with DumaTherm Manager v4.17 (Gerhardt, Königswinter, Germany) software. All experiments were performed in two duplications of the same sample.

### 3.7. Targeted Analysis of Monomers and Oligomers

The quantification was based on a modified method of the fast GC method reported by Tsochatzis et al. [1] using styrene and PS oligomer standards, which were prepared in CHCl_3_, and external calibration was applied, according to a previously reported method [1]. The organic CHCl_3_ extracts were filtered with PTFE 0.22 μm filters, and a volume of 1 μL was injected by the GC-TOF-MS system in split-less mode into an Agilent Technologies 7890B gas chromatography system coupled to an Agilent Technologies 7200 Accurate-Mass Q-ToF mass Spectrometer (Agilent Technologies, Waldbronn, Germany). Separation of the metabolites was performed on an HP-5MS capillary column (30 m, 0.250 mm i.d., 0.25 μm film thickness, Agilent Technologies). The ion source temperature was set to 230 °C, and the MS analysis was performed in scan mode with a quadruple temperature of 150 °C and a fragmentation voltage of 70 eV (pressure of 21.4 psi, a gas saver of 20 mL/min and 3 mL/min purge flow). The GC oven temperature program followed the temperature program previously reported in [1]. Briefly, an isotherm of 60 °C was held for 2 min, followed by a temperature ramp of 10 °C/min up to a temperature of 320 °C that was held for 5 min. The total analysis time was 34 min.

### 3.8. Data Analysis and Statistical Analysis

Statistical analysis and pairwise analysis were performed by one-way ANOVA using RStudio (RStudio Team, 2020). On this basis, the identified components were analyzed by principal component analysis (PCA) to assess the discrimination between the different feeding strategies, performed with SIMCA 17.0.1 software (Umetrics, Umea, Sweden).

## 4. Conclusions

The gut microflora of *T. molitor* varied depending on the PS:bran ratio and the presence or absence of H_2_O supplied with the diet. The microbiome data indicated that three bacteria, *Erwinia olea*, *Lactococcus lactis* and *Lactococcus garviae*, were more abundant in the gut samples of *T. molitor* fed with PS compared with the control, rendering them as three potential candidates for further studies on their role in the biodegradation of PS. No degradation products were observed to be accumulated in insects’ biomass, compared to frass, where PS monomers (styrene) and oligomers such as 1,1-diphenyl cyclobutene, 2,4-diphenyl-1-butene, trans-1,2-diphenyl cyclobutene, 1,3,5-trihenylcyclohexene, 1a-phenyl-4-(1-phenylethyl)-1,2,3,4-tetrahydronaphthalene and 1e-phenyl-4-(1-phenylethyl)-1,2,3,4-tetrahydronaphthalene were quantified. A higher number of residues were identified in the diet with the low bran:PS ratio due to non-efficient degradation of the polymer. The diets were well clustered, showing differences in protein residues, frass nitrogen content, growth and microbiota. These findings could indicate appropriate biodegradation, and they were correlated with the reported microbiome. The presence of water certainly affected both PS degradation and the observed microbiome, while it facilitated higher PS degradation rates and lower formation of excreted PS monomers and oligomers. However, when comparing our data with those already present in the literature, it can be concluded that microbiome patterns may completely differ among studies within the same gut host, and that the role of the whole microbiota and the potential interactions existing between the bacterial communities living in the digestive tract of *T. molitor*, as well as their role in PS biodegradation, are not entirely understood. As a result, further research is needed.

## Figures and Tables

**Table 1:** Identified bacterial counts in the digestive tracts of mealworm larvae after degrading PS following different feeding protocols.

Family	Genus	Species	Control	Diet 1 (Bran: PS 4:1)	Diet 2 (Bran:PS 20:1 + H_2_O)	Diet 3 (Bran:PS 20:1)
*Spiroplasmataceae*	*Spiroplasma*	*sp.*	68,567	55,880	66,520	77,787
*Enterobacteriaceae*	*Klebsiella*	*pneumoniae*	31,778	12,065	3602	877
*Enterobacteriaceae*	*Erwinia*	*oleae*	2038	10,977	8787	3485
*Enterococcaceae*	*Enterococcus*	*faecium*	602	0	4689	474
*Streptococcaceae*	*Lactococcus*	*lactis*	217	425	1542	253
*Streptococcaceae*	*Lactococcus*	*garvieae*	114	348	390	449
*Leuconostocaceae*	*Weissella*	*cibaria*	210	7	986	25
*Enterococcaceae*	*Enterococcus*	*mundtii*	0	968	0	0
*Clostridiaceae-1*	*Clostridium*	*sp.*	198	193	103	389
*Flavobacteriaceae*	*Chryseobacterium*	*sp.*	122	157	88	201
*Pseudomonadaceae*	*Pseudomonas*	*sp.*	19	53	0	45
*Ruminococcaceae*	*Faecalibacterium*	*prausnitzii*	13	19	0	6
*Enterobacteriaceae*	*Proteus*	*sp.*	21	5	8	0
*Xanthomonadaceae*	*Stenotrophomonas*	*Rhizophila*	0	14	0	14
Total *n* of reads			103,953	81,188	86,776	84,133

**Table 2:** Identified fungi counts in the digestive tracts of *T. molitor* after degrading PS following different feeding protocols.

Family	Genus	Species	Control	Diet 1 (Bran:PS 4:1)	Diet 2 (Bran:PS 20:1 + H_2_O)	Diet 3 (Bran:PS 20:1)
*Sporidiobolaceae*	*Sporobolomyces*	*roseus*	0	0	0	36
*Pleosporaceae*	*Alternaria*	*alternata*	0	0	0	27
*Trichocomaceae*	*Aspergillus*	*versicolor*	0	0	0	27
*Pleosporaceae*	*Pithomyces*	*chartarum*	16	0	0	0
*Tremellaceae*	*Cryptococcus*	*carnescens*	5	0	0	10
*Trichocomaceae*	*Aspergillus*	*sp.*	0	13	0	0
*Davidiellaceae*	*Cladosporium*	*sp.*	0	0	0	9
*Davidiellaceae*	*Cladosporium*	*herbarum*	0	8	0	0
*Malasseziaceae*	*Malassezia*	*sp.*	0	0	8	0
*Pleosporaceae*	*unclassified (F: Pleosporaceae)*	*sp.*	0	6	0	0
*Trichocomaceae*	*unclassified (F: Trichocomaceae)*	*sp.*	0	6	0	0
*unclassified (Dothideomycetes)*	*unclassified (C:Dothideomycetes)*	*sp.*	5	0	0	0
*Saccharomycetaceae*	*Candida*	*albicans*	0	0	0	5
Total *n* of reads			26	33	8	114

**Table 3:** Identification and quantification of formed PS monomers and oligomers in insects’ frass.

Target Analytes	Average Mass Fractions in Frass (μg/mg) (±SD)			
	Control	Diet 1 (Bran:PS 4:1)	Diet 2 (Bran:PS 20:1 + H_2_O)	Diet 3 (Bran:PS 20:1)
1,1-diphenyl ethylene	0	0.016 (±0.002)	0.008 (±0.001)	0.007 (±0.002)
Styrene	0	0.005 (±0.001)	0.003 (±0.001)	0.002 (±0.001)
2,4-diphenyl-1-butene	0	0.014 (±0.001)	0.004 (±0.001)	0.004 (±0.001)
trans-1,2-diphenyl cyclobutane	0	0.013 (±0.002)	0.005 (±0.002)	0.003 (±0.001)
1e-tetrealin *	0	0.006 (±0.001)	0.004 (±0.001)	0.005 (±0.001)
1a-tetralin **	0	0.014 (±0.002)	0.006 (±0.001)	0.005 (±0.001)
Acetophenone	0	0.180 (±0.020)	0.070 (±0.014)	0.050 (±0.012)
2,4-di-tert butyl phenol	0	0.100 (±0.010)	0.050 (±0.011)	0.040 (±0.010)
2,4,6-triphenyl-1-hexane	0	0.173 (±0.019)	0.051 (±0.013)	0.030 (±0.011)

Note: SD = standard deviation. * Full chemical name: 1e-phenyl-4-(1-phenylethyl)-1,2,3,4-tetrahydronapthalene. ** Full chemical name: 1e-phenyl-4-(1-phenylethyl)-1,2,3,4-tetrahydronapthalene.

**Table 4:** Composition of the barley bran used, as provided by the feed supplier.

Component	Protein (%)	Starch (%)	Fat Content (%)	Soluble Fibers (%)	Insoluble Fibers (%)	Ash Content (%)
**Amount**	14.7	22.0	4.5	2.8	43.2	5.6

**Table 5:** Feeding diets, including amounts of nourishment and PS plastic, provided to the larvae for each biodegradation experiment.

	Feeding Diets			
	Control	Diet 1	Diet 2	Diet 3
Rolled barley (g) *	20.0 ± 0.01	3.99 ± 0.06	20.1 ± 0.03	20.1 ± 0.03
Polystyrene (PS) (mg)	0.0	1000 ± 10	980 ± 16	1015 ± 17
Water (mL) **	0	0	1.5	0

* The composition of the barley bran is presented in Table 4 (Section 3.1). ** Equal volume was administered at the beginning of the experiment and every 4th consecutive day.
